# Impact of the COVID-19 pandemic on young adults’ mental health and beyond: a qualitative investigation nested within an ongoing general population cohort study

**DOI:** 10.1007/s00127-024-02659-5

**Published:** 2024-04-05

**Authors:** Anna Wiedemann, Peter B. Jones, Anne-Marie Burn

**Affiliations:** 1 Department of Psychiatry, University of Cambridge, Douglas House, 18B Trumpington Road, Cambridge, CB2 8AH UK; 2 Cambridgeshire and Peterborough NHS Foundation Trust, Cambridge, UK; 3 Applied Research Collaboration, National Institute for Health Research, East of England, Cambridge, UK

**Keywords:** COVID-19, Pandemic, Young adults, Mental health, Qualitative

## Abstract

**Purpose:**

Initial discussions about the COVID-19 pandemic often overlooked its impact on young adults. By employing a qualitative approach nested within an ongoing general population cohort study, we seek to fill a gap in the literature by providing insights into the longer-term impact on this demographic.

**Methods:**

Data collection involved the use of in-depth semi-structured interviews. Using a pre-determined sampling frame, we purposively recruited 30 participants based on age, gender, ethnicity, and deprivation from the Neuroscience in Psychiatry Network (NSPN). The NSPN cohort, established in 2012, consists of 2403 young people aged 14–24 at baseline, recruited from Greater London and Cambridgeshire. Interviews were conducted in Autumn 2022; data were analysed using the framework method.

**Results:**

Participants were on average 28 years old (SD = 3 years, range 24–34 years; 53.3% female). The sample comprised individuals from diverse ethnic backgrounds, with 40% from non-White ethnic groups. Many young adults reported profound personal growth and a stronger sense of resilience, a perception observed across varying levels of anxiety or depression. Nevertheless, we observed substantial disruptions to their personal and professional lives such as returning to their parents’ homes, often deferring other life plans, lacking mental health support, and encountering significant career challenges.

**Conclusion:**

Our findings highlight the complexity of pandemic impacts, demonstrating the need for supportive policies and further research to understand the circumstances under which genuine personal growth occurs, whether it is enduring or transient, and which factors influence it.

**Supplementary Information:**

The online version contains supplementary material available at 10.1007/s00127-024-02659-5.

## Introduction

The COVID-19 pandemic brought about unprecedented adjustments across various societal domains, profoundly shaping the experiences of young adults. It affected a substantial phase in their lives that is characterised by change and personal development. For many, young adulthood is a period of personal growth and pivotal life decisions, particularly in terms of education and occupational pursuits [1]. However, it is also a period of heightened vulnerability to the onset of serious mental health conditions with potential detrimental effects on education, earning potential, and social functioning [2]. Disruptions caused by the pandemic may have the potential to create enduring mental health challenges that persist beyond the subsiding of the pandemic itself. Over the past few years, young adults have faced significant challenges, particularly in terms of economic and employment downturns. For instance, educational interruptions and job insecurity have exacerbated uncertainties and anxiety amongst this age group [3, 4]. These experiences have likely been disproportionately felt by individuals already exposed to socio-economic marginalisation and deprivation [5].

Despite lower susceptibility to severe physical health consequences associated with COVID-19, young adults have been particularly affected by the implementation of transmission mitigation strategies and government pandemic responses [6–8]. In late April 2020, for instance, a quarter of the population in the United Kingdom (UK) experienced significant mental distress, with the steepest increase compared to pre-pandemic levels observed in those aged 18–34 [6]. Evidence further suggests temporary fluctuations in loneliness that coincided with the implementation of lockdown restrictions in this age group [9]. Perhaps predictably, the pandemic has had a considerable impact on their social connectivity, resulting in the loss of social networks, increased social pressure and stress in maintaining digital relationships, the distancing of peripheral friendships, yet the simultaneous strengthening of close friendships [10]. A recent systematic review focusing on mental health changes in children and young people before and during the COVID-19 pandemic identified a longitudinal decline in mental health, evidenced by increased depression, anxiety, and psychological distress [11]. Another review, however, suggests the pandemic has had a more nuanced effect on population mental health than previously thought, with limited substantial changes observed overall [12]. Despite this evidence, research specifically focusing on young adults remains scarce, with existing studies predominately quantitative. However, due to the unique complexities associated with young adulthood, there is a need for a qualitative inquiry that delves into their thoughts, emotions, and reflections, complementing the quantitative assessments.

The aim of our study was to primarily investigate the impact of the COVID-19 pandemic on young adults’ mental health and wellbeing, whilst also considering the influence of interpersonal relationships, coping and adaptation processes, and occupational experiences. We hope that through capturing their voices, our qualitative study will provide valuable insights and deepen our understanding of the long-term effects of the pandemic.

## Methods

Data collection involved the use of in-depth semi-structured interviews. We followed the guidelines outlined in the Consolidated Criteria for Reporting Qualitative Research (COREQ) checklist to ensure throughout reporting of our findings [13]. Ethical approval was granted by the Cambridge East Research Ethics Committee under REC 16/EE/0260.

### Setting

Participants were recruited through the Neuroscience in Psychiatry Network (NSPN), a longitudinal cohort study investigating developmental changes and psychiatric disorders through comprehensive data collection [14]. The cohort, established in 2012, consists of 2403 young people aged 14 to 24 at baseline, recruited from the general population across Greater London and Cambridgeshire. Multiple follow-up assessments have been conducted, including two pandemic-specific follow-ups launched in May 2020 and July 2022 [15]. Interviews were conducted during the second COVID-19 follow-up in October 2022, capturing the experiences and perspectives of participants in their early 20 s to 30 s.

### Research team

Our research team comprised two female chartered psychologists (AW, AMB) and one male psychiatrist (PBJ), all of whom brought diverse expertise to the study, albeit primarily White and of middle socio-economic backgrounds. This composition reflects limited demographic diversity, yet the range of ages within our team, particularly with the lead author (AW) close to the age of participants, enriched our interpretive approach. As the most experienced qualitative researcher, AMB provided guidance and supervision throughout all stages of the study. AW assumed the lead in conducting the interviews and developed the topic guide as well as the coding framework with input from AMB. AW further led the analysis phase with collaborative contributions from both AMB and PBJ in the interpretation and reporting of the study.

### Participant recruitment

Participants for this study were selected from the NSPN database, with a focus on individuals who had participated in previous assessments and who had explicitly granted consent to be contacted for further research. Using a pre-determined sampling frame to ensure diversity, we purposively recruited participants based on age, gender, ethnicity, and deprivation. By incorporating a wide range of socio-economic backgrounds, we sought to capture a broader spectrum of experiences and perspectives on the pandemic’s impact, particularly given the potential for more pronounced effects amongst lower socio-economic groups. Invitations were sent in waves via email. Deprivation was assessed by the Index of Multiple Deprivation (IMD; [16]) via postcode at baseline only. The IMD ranks every small area in England from most deprived to least deprived whereas relative deprivation is most commonly described in deciles with the lowest decile representing the most deprived 10% of areas in England. As individuals from the lowest deciles were underrepresented in the NSPN cohort and had lower response rates to interview invitations, we specifically invited more individuals from these backgrounds and sent up to three reminders to enhance participation. This effort was part of our strategy to include an equal number of participants per decile, ultimately determining our final sample size.

Invitations were sent out between 15 September and 17 October 2022, resulting in a total of 86 approached participants. Among these, 46 expressed an interest in participating, and ultimately, 30 participants were interviewed, with the remaining participants placed on a waiting list for potential cancellations. All participants received a participant information sheet and were encouraged to contact the researchers for any clarifications. Written informed consent was obtained from all participants beforehand. At the start of the interview, participants were provided with comprehensive information regarding the research project's objectives. Upon completion of the interview, participants received a £25 multi-retailer gift voucher as an expression of gratitude for their time.

### Data collection

Data collection took place between 29 September and 25 October 2022 via video call. Interviews followed a topic guide that was developed based on emerging findings from the first NSPN COVID-19 follow-up [17]. Our topic guide underwent pilot testing that involved initial feedback from young adult volunteers on its clarity and relevance, followed by a test interview for detailed feedback. This process led us to refine some of the questions; we also added potential prompts to enhance response depth. The topic guide can be found in the Supplementary Materials 1. Briefly, the interviews primarily focused on the impact of the pandemic on mental health and wellbeing, whilst also exploring topics such as interpersonal relationships, coping mechanisms, adaptive processes, and occupational experiences. These topics were primarily explored with an interest in understanding how they influenced, and were affected by, participants’ mental health. Participants were instructed to ensure privacy and minimal interruptions during the interview. All interviews were audio recorded and additional notes were taken after each session. Recordings were subsequently transcribed verbatim by an external transcription company and de-identified to protect participants’ confidentiality. Transcripts were not returned to participants for comments or corrections. The average interview duration was 38 min (range 27–60 min), excluding the opening remarks and final segment.

### Data analysis

Transcripts were imported into NVivo 12 software [18] for analysis using the framework method [19]. The thematic framework was developed by AW, who engaged in an in-depth review of five transcripts and consulted the topic guide for guidance. AMB also coded a distinct subset of two transcripts and provided feedback on the initial thematic framework. Following this, AW analysed an additional five transcripts, incorporating further refinements. This updated framework was then shared with the entire research team to ensure comprehensive coverage, coherence, and clear distinctions between themes. Subsequent to this only minor adjustments were necessary which were discussed with the research team as they arose. The final thematic framework, including a detailed summary of each code to reflect the data captured, can be found in the Supplementary Materials 2. A simplified overview is provided in Table 1. Next, AW proceeded to create a matrix framework, organising the data by codes (columns) and cases (rows). The matrix cells were then populated with summarised data and verbatim from the transcripts, accompanied by analytical notes (‘charting’). This approach facilitated a detailed and accessible overview of the qualitative dataset. To guide the subsequent analysis, summaries of the data under each theme were developed and where appropriate further sub-themes were identified.

**Table 1:** The coding framework

Coding framework
Domain 1: pandemic general impact
Initial impact and response
Impact on cultural and societal behaviours and values
Domain 2: social networks & dynamics
Family life and relationship impact
Friendship impact
Domain 3: mental health & wellbeing
Mental health challenges
Access to mental health support and therapy
Coping strategies and adaptive pursuits
Reflective growth and resilience
Domain 4: work & education
Work challenges and ramifications
Workplace culture and support
Working from home
Education and teaching

Further details on the coding framework can be found in the supplementary materials where each code has been summarised concisely to describe the specific data it aims to capture. Please note that within the coding framework, additional labels titled “other” were assigned to each domain. Whilst not listed in this table, these labels are elaborated upon in the supplementary materials

### Data linkage

All participants provided consent for linking their interview data with their existing NSPN records. For descriptive purpose, we report summary statistics for anxiety and depression, as assessed during the second NSPN COVID-19 follow-up launched in July 2022. Anxiety was measured using the Generalised Anxiety Disorder Assessment (GAD-7; [20]) whilst depression was measured using the Patient Health Questionnaire (PHQ-9; [21]). Both scales use 4-point Likert responses, with scores ranging from 0 to 27 (PHQ-9) and 0 to 21 (GAD-7). Clinical cut-offs are ≥ 10 for depression and ≥ 8 for anxiety [22]. Additionally, we report summary statistics for loneliness, assessed using the UCLA 3-Item Loneliness Scale, with scores between 6 and 9 indicating loneliness [23].

## Results^1^

**Fig. 1:**
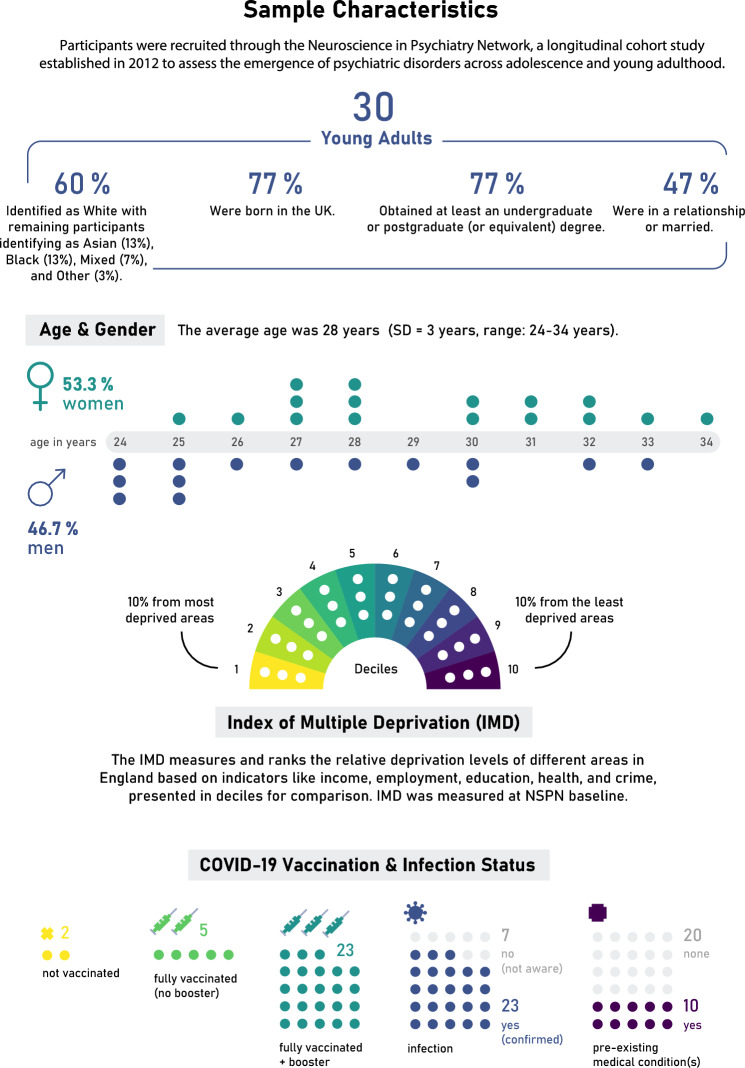
Sample characteristics of NSPN COVID-19 interview participants

We recruited a sample of 30 young adults for the study, with 53.3% identifying as female and 46.7% as male. The average age was 28 years (SD = 3 years, range 24–34 years). The sample comprised individuals from diverse ethnic backgrounds, with 40% from non-White ethnic groups. Sample characteristics are presented in Fig. 1. Mental health data collected during the latest NSPN COVID-19 survey conducted from July to October 2022, showed, amongst those interviewed, the average GAD-7 score was 5.9 (SD = 4.0) and the PHQ-9 score was 6.5 (SD = 4.8). Notably, 17% of interview participants reported anxiety scores and 23% depression scores that met National Health Service (NHS) guidelines for clinical treatment [19]. Additionally, using the UCLA Loneliness Scale, 50% of participants crossed the threshold for experiencing loneliness.

Our findings, primarily centred on the impact of the COVID-19 pandemic on mental health and wellbeing, are organised into four key domains. These domains explore the pandemic’s influence on mental health as well as its effects through various life aspects such as social networks and dynamics or the impact on work and education.

### Domain 1: pandemic general impact

#### Initial impact and response

Initially, most participants reported being unconcerned about COVID-19. However, their perception changed after personal experiences such as hearing about people they knew passing away or being hospitalised. Concerns were predominately centred not around catching the virus but transmitting it. Simultaneously, some expressed contradicting feelings of anxiety combined with a short-lived excitement: *“There was a slight element of…this is relatively new. It’s…like when there’s a power cut and everyone starts lighting candles, it’s…slightly exciting for a short period of time. But I think there was a lot of anxiety”* [P17, Male, 27 years].

#### Impact on cultural and societal behaviours and values

Many participants described feeling pessimistic about the future, criticising the government's lack of seriousness in handling the pandemic. Additionally, some noted an increase in political division within the country: *“I would say there's just a lot more political division. So, when I meet new people, sometimes I find that they might disagree with me on certain topics and that makes it a bit awkward”* [P26, Female, 28 years]. Nonetheless, many felt a positive shift in some societal attitudes, particular in relation to mental health: *“I found that when I started talking about it [mental health], …people started talking about it to me more. It was like this wonderful door had been opened”* [P16, Male, 30 years].

### Domain 2: social networks & dynamics

#### Family life and relationship impact

Almost half of participants stayed or moved back in with their parents, often delaying other life plans. Many were still living with their parents at the time of interview, i.e., more than two years after the initial outbreak. Many participants felt they grew closer to family members and partners. For some, the constant reminder of mortality strengthened bonds, but also facing challenges together was often described as solidifying relationships: “*So, it's definitely made me closer to my partner, because that was quite a big test suddenly being home 24/7”* [P25, Female, 28 years]. Nevertheless, some participants experienced heightened conflicts: *“I actually share a room with my older brother…I did start to feel like a lot of like bitterness like a bit of hatred inside”* [P20, Male, 24 years]. Some participants further faced family conflicts over differing views on the COVID-19 vaccine, for some, leading to avoiding the topic or hiding their vaccination status: *“When the vaccines came out, that's when we started to have some issues because I was pro-vaccine. A lot of them weren't…we don't discuss it now. Yes, it bothered me a lot when some of them refused to take the vaccine”* [P26, Female, 28 years].

#### Friendship impact

Most participants reported feeling a stronger bond with close friends but simultaneously distancing themselves from more peripheral friends: “*I think the key that came out of lockdown was that I kept the friends that were willing to stick by me and didn’t with the others. And I think that’s very good”* [P07, Male, 25 years]. Some participants mentioned that not being able to share important milestones with their friends during the pandemic made it harder to maintain their friendships and regain their pre-pandemic closeness: *“So a lot of my friends matured to a point where they were concentrating on their own families or starting families, which was really lovely, but obviously we couldn't see or be a part of that journey.”* [P29, Female, 30 years].

### Domain 3: mental health & wellbeing

#### Mental health challenges

Particularly during the early stages of the pandemic, many participants struggled with increased feelings of anxiety, isolation, and uncertainty. Those with pre-existing mental health conditions experienced amplified struggles: *“I was breaking down in every possible way that I can think of. I would feel constantly down. I would feel let down and lonely and unwanted, unloved”* [P05, Female, 27 years]. Additionally, those looking after someone with mental health problems reported a significant strain on their own wellbeing: “*So, he [partner] was at that stage where he was one of those people that was just in bed and couldn't really do anything…he was not very much in survival mode. So, there was a huge demand on me for about a year”* [P21, Female, 28 years].

Furthermore, some participants lost loved ones during the pandemic and sought therapy. Others reported being deeply affected by restrictions preventing hospital visits: *“So, that was…the culmination of 15 months of not seeing someone, one of the most important women in your life and then she’s got stage 3 cancer…that’s nuclear at that point. And 15 months of not seeing your own mother, it feels like I’ve been robbed of that time by something that’s not in my control”* [P16, Male, 30 years].

Moreover, participants described varied reactions to testing positive for COVID-19, a few reported feeling initial intense fear and anxiety, especially those with pre-existing health conditions: *“The first couple of days it hit me hard. Thinking, what if I don’t make it through this?”* [P13, Male, 32 years]. Even though the majority did not report experiencing prolonged symptoms after their infection, those who did, faced significant consequences: *"Foggy head…if you haven’t slept well and you wake up and your head feels so heavy, it’s just kind of like that all the time”* [P16, Male, 30 years].

#### Access to mental health support and therapy

Those wanting to access mental health support faced significant barriers, with almost everyone facing difficulties securing services through the NHS due to unresponsiveness, long waiting lists, and appointment challenges. Some participants felt overwhelmed and did not even attempt to seek help, anticipating these issues: *“I didn’t really try to be honest…because the waiting lists for the NHS counselling is just ridiculous anyway and I think, you know, I would prefer that in person contact”* [P16, Male, 30 years]. Many reported that the available mental health resources often did not suit their needs, and some highlighted a gap in semi-urgent mental health care provision. A few participants reported finding support through charities, but felt it was not sufficient. One participant who could afford private services reported a smoother process in accessing care.

Those accessing therapy, primarily outside the NHS, had positive experiences, with therapy aiding recovery from depression, anxiety, and fostering a greater sense of self-awareness. Individuals noted the convenience and privacy of phone and online sessions as beneficial during the pandemic, as these formats allowed them to express themselves freely within the comfort of their own homes: “*I think it helped me to not know what my therapist looked like…I felt like I was able to be in my home and I was speaking to somebody in my ears, but I wasn't having to—so, I could, like, cry and whatever else I needed to do”* [P04, Female, 27 years].

#### Coping strategies and adaptive pursuits

Participants adopted various coping strategies, for instance, many found comfort in nature, especially through walking and gardening: *“It's something that I was seeing it as a bit pointless before, but now I love it, I feel so good and refreshed after a walk”* [P03, Male, 30 years]. Exercise further emerged as an important coping mechanism: “*I already knew pre-pandemic that the number one thing for me individually, in terms of maintaining a healthy mindset about things, is exercise*” [P27, Male, 26 years]. Many reported seeking distraction through, for instance, activities such as drawing, reading, or gaming: “*I didn't start drawing because I thought it would help but I was really surprised that it was helping in the end”* [P18, Female, 34 years]. Most participants further described that seeking social connection, including those with pets, played a crucial role in maintaining their emotional wellbeing. Even though quite a few recognised their attempts to maintain a positive outlook, a small number of individuals resorted to maladaptive coping strategies, including turning to substances like alcohol and nicotine.

#### Reflective growth and resilience

Many participants described personal growth and increased resilience. Even though some reported facing initial difficulties, many felt that the pandemic allowed them to reflect and grow stronger: “*I feel like I’ve generally been growing and finding out what I want to do a bit more, which is good”* [P06, Male, 29 years]. Both personal growth and resilience has been descripted by those with and without self-reported anxiety or depression as measured by the GAD-7 and PHQ-9. For many, the pandemic heightened their adaptability, especially when situations did not unfold as planned. Quite a few participants further described paying more attention to their mental health and understanding their reactions to stress: *“If I was struggling [pre-pandemic] I just thought it was a part of my day or a part of my week, my month. Whereas now if I’m struggling, I can be a bit more active in improving it”* [P09, Female, 31 years].

### Domain 4: work & education

#### Work challenges and ramifications

The pandemic significantly disrupted career trajectories, particularly for young professionals, with many graduates reporting facing a tough job market and missing out on opportunities: *“I was applying for retail jobs, I was applying for jobs in my field, I was applying for literally anything that was available, and it was just a completely shoddy market…it took a bit of a hit as well in terms of my mental health because it was just constant onslaught of rejections”* [P23, Male, 24 years]. Conversely, some reported pandemic-related changes in their line of work as positive. The lockdown allowed many to introspect and reconsider their career choices, leading some to shift professions, prioritise their mental wellbeing, or acknowledge personal growth despite challenges: *“The pandemic, particularly my work, gave me an opportunity to act up as deputy service lead and grow enough to get this position and I don't know if I'd have done that otherwise”* [P21, Female, 28 years].

Nonetheless, for some, the pandemic caused significant socio-economic disruptions. One participant, for instance, described the stress of losing their sense of control when all their work engagements were cancelled overnight. Even though being furloughed (i.e., being placed on temporary leave as part of the UK Coronavirus Job Retention Scheme) was often described as a big relief, those affected expressed heightened concerns about job insecurity. Additionally, some struggled with the mental consequences of transitioning from an intense full-time job to suddenly doing nothing: *“I was on furlough for eight months…it was almost a numbing sensation. You’d drive so hard, and you’d work 70-h weeks…it was hard to understand and accept”* [P07, Male, 25 years]. Overall, socio-economic hardship was more frequently reported by participants from lower socio-economic backgrounds.

#### Workplace culture and support

Many participants felt isolated, especially new graduates entering their first jobs: *“I was quite lonely in my first job; I was only by myself. I never got to see anybody”* [P19, Female, 25 years]. Whilst some reported positive support structures, many experienced a lack of effective leadership, with delayed responses to concerns and perceived inaccessibility of management. A minority did feel fully supported, however, crediting their management and colleagues for their emotional wellbeing during this period.

#### Working from home

Participants’ experiences working from home during the pandemic varied largely, spanning from strong aversion to enthusiastic approval of a fully remote setup. Many appreciated the enhanced work-life balance, reporting increased productivity and better mental wellbeing: *“I found that I worked much better at home than I do in the office because I can shut myself away and really focus and concentrate”* [P25, Female, 28 years]. Some were neutral, recognising the benefits but missing the office’s social aspect, and worried about professional development: *“I found that my ability to do work wasn't hindered…but my ability to professionally develop…I felt that if they [colleagues] were there I’d be able to develop more quickly and get more engaging work”* [P27, Male, 26 years]. In contrast, others struggled with working from home, finding it hard to separate work from personal life, feeling isolated, or experiencing monotony, often affecting their mental wellbeing.

#### Education and teaching

Participants who were students experienced significant academic setbacks and feelings of loneliness. Many felt deprived of their university experience, missing out on internships, placements, and essential social interactions, leading to frustration, isolation, and motivational challenges. Many reported that working from home made studying monotonous and draining. Those enrolled in professional training programmes questioned the quality and effectiveness of their pandemic-altered education, raising doubts whether their training met pre-pandemic standards: *“I feel like I missed out on some stuff as well, it was a very practical course, and we sort of had to muddle through and do some of that stuff online…I’ll say this diminished the quality of therapists qualifying at this time, potentially”* [P30, Male, 33 years].

## Discussion

Initial discussions about the potential impact of the COVID-19 pandemic often overlooked its implications for young adults, a crucial life stage characterised by change and personal development, even without the added challenge of a pandemic. Our study sought to understand how this age group experienced the pandemic, specifically how it affected their mental health.

Throughout our analysis a recurring theme of profound reflection and personal growth emerged as a key insight cutting across various domains and themes of our coding framework. For many young adults, the pandemic acted as a catalyst, prompting them to reflect on their own mental health and wellbeing, often leading to shifts in their priorities in this area. This extended into their professional lives, often triggering career reassessments and shifts in behaviour such as ensuring a healthy work-life balance during and post-pandemic. Many reported emerging from the pandemic feeling stronger and more resilient. Whilst our findings contribute to a rather small body of literature, they closely align with existing research on the positive and transformative effects arising from the COVID-19 pandemic [24–27], a concept frequently associated with post-traumatic growth in the broader psychiatric literature [28]. However, it is important to emphasise that the pandemic itself should not be universally classified as inherently traumatic. Nonetheless, our findings suggest that it has instigated similar effects to those often observed in response to a wider range of crises, including natural disasters, conflict, physical illness, divorce, or bereavement [29–34]. Such challenges often stimulate personal growth, and it is not uncommon for individuals to emerge from these crises with enhanced social and personal resources, along with new coping skills that, in our study, were generally healthy and adaptive [31]. Yet, among the limited number of pandemic-specific studies assessing positive outcomes, one in particular found that personal growth can assume an illusory nature [24]. In this study, some individuals used it as a self-deceptive strategy to persuade themselves that they are coping more effectively than was actually the case. In our study, participants reported personal growth irrespective of their self-reported levels of anxiety or depression, suggesting that experiencing personal growth did not necessarily equate to the absence of mental ill-health. The distinction between perceived personal growth and actual mental wellbeing, however, was not directly assessed in our interviews. This highlights the need for additional research to fully understand the circumstances under which genuine personal growth occurs, whether it is enduring or transient, and which factors influence it. Such future investigations should consider both environmental and personal resources to better grasp the positive outcomes during pandemics.

Our analysis further revealed substantial disruptions to the personal and professional lives of many young adults, with potential implications for their transition to adulthood. Notably, almost half of the participants returned to or lived with their parents during the pandemic, often deferring other life plans. Many continued in these living arrangements beyond their initial expectations. Whilst many reported improved family connections, some also experienced heightened conflicts and strained relationships as a result. These findings are consistent with existing research, which indicates that whilst returning to the parental home during the pandemic was a positive experience for many young adults, for some, living with their parents gave rise to additional difficulties which negatively affected their mental health [35–37]. Furthermore, participants experienced significant disruptions to their professional and educational journeys. Recent graduates, in particular, reported facing a challenging job market and missing out on important career opportunities. For some, the pandemic led to considerable socio-economic disruptions and hardship. These were more frequently reported by participants from lower socio-economic backgrounds, aligning with expectations of the disproportionate impact of the pandemic on these groups. Those still in education encountered academic setbacks, feeling deprived of their university experience, leading to frustration, isolation, and motivational challenges. Overall, these findings suggest that the COVID-19 pandemic has intensified the inherent instability of young adulthood. The accumulation of stressors during this period may influence long-term mental health outcomes, aligning with research indicating that adverse experiences in earlier life stages and adulthood increase the risk of depression later in life [38]. Whilst our findings indicate substantial resilience within this population, assessing the enduring consequences of pandemic-related disruptions on the development of young adults as they progress into full adulthood is therefore crucial.

Continuing our analysis, a significant concern arising from our findings is the clear lack of mental health support for those in need. Participants faced significant barriers when trying to access mental health services through the NHS, often due to unresponsiveness, long waiting lists, and appointment challenges. Beyond, once offered help, many perceived the services provided to be insufficient and poorly aligned with their needs. Unfortunately, these findings may not be surprising, as recent reports from the National Audit Office have also emphasised the insufficiency of mental health services in the UK, particularly given the increased demand for such services due to the COVID-19 pandemic [39]. Given the challenges in accessing mental health services, remote therapy emerges as one potential solution to improve reach. Yet, despite its convenience being valued by some of our study participants, the generally limited availability of support restricts our ability to fully evaluate its benefits. Overall, our findings highlight the growing gap between the mental health needs of the population and the quality of provision, requiring attention and improvements to address these fundamental issues. Importantly, our study further showed that lack of adequate support was not limited to young adults requiring mental health assistance but also affected young graduates and those commencing their professional journeys. To address the disruptions caused by the COVID-19 pandemic, our findings highlight the urgency of implementing comprehensive policies that support young adults, particularly those facing mental health challenges. Whilst pointing to the necessity of such policies, we acknowledge the need for further research to fully grasp the long-term impacts and to develop policy responses tailored to the evolving needs of this age group.

### Strength and limitations

Our study offers important insights into the challenges faced by young adults during the COVID-19 pandemic. A strength of this study is that our sample was diverse in terms of their socio-economic backgrounds, ethnicities, age, and gender which provided a range of perspectives and experiences. The ability to sample from an existing, large epidemiologically-principled cohort of young adults followed over a decade, with the possibility of linkage to prior data is an unusual design. However, certain limitations should be acknowledged. More than two-thirds of our sample held undergraduate or postgraduate degrees, which mirrors the broader NSPN cohort’s over-education issue [15], and therefore our findings may not be applicable to young adults without a higher education. Additionally, our study’s context within a high-income setting with available pandemic support measures may limit the applicability of our findings to settings in low-and-middle-income countries, where different socio-economic challenges and support systems could have altered the pandemic’s impact on young adults. Furthermore, approximately one third of recruited participants worked in the healthcare sector, and this may have influenced their decision to take part in the research and their views may not reflect others who do not work in healthcare. Finally, we acknowledge the presence of thematic overlap in our analysis and want to highlight that our coding framework, which we believe to be robust and effective in presenting the findings, has undergone extensive team discussions. However, we also recognise the potential validity of alternative interpretations.

### Conclusions

Many young adults perceived substantial personal growth during the COVID-19 pandemic, prompting reflection on mental health, shifting priorities, and triggering career reassessments. They reported emerging from the pandemic feeling stronger and more resilient. Nevertheless, we observed significant disruptions to their personal and professional lives, intensifying the inherent instability of this developmental period. Many struggled with increased feelings of anxiety, isolation, and uncertainty, particularly during the early stages of the pandemic. Those with pre-existing mental health conditions experienced amplified struggles. Moreover, many reported moving back in with their parents, often deferring other life plans. Recent graduates faced a challenging job market, and those still in education encountered academic setbacks, feeling deprived of their university experience. The lasting impact of these disruptions on the transition of these young adults into full adulthood remains uncertain and requires further investigation. Finally, and most significantly, our findings highlight the need for accessible and well-aligned mental health support, as the current barriers and insufficiencies within NHS services are concerning and must be addressed promptly.

## Supplementary Information

Below is the link to the electronic supplementary material.

Supplementary file1 (PDF 166 KB)

## Data Availability

To maintain participant confidentiality and adhere to the principles of informed consent and ethical approvals granted for the study, the interview transcripts are not publicly accessible in order to prevent the identification of individual participants. In general, however, de-identified *quantitative* data from NSPN assessments is fully available to the research community and can be requested and downloaded through the Open:NSPN portal following this link: https://nspn.org.uk/.

## Footnotes

1 Given the extensive nature of the interviews, we present a condensed results section here, focusing on novel insights and the most impactful themes. Broader but well-documented phenomena and less frequent observations are detailed in Supplementary Materials 3. We recommend accessing the extended results if a more in-depth understanding is wanted.
