# Supplementary material for: Impact of the COVID-19 pandemic on young adults’ mental health and beyond: a qualitative investigation nested within an ongoing general population cohort study
Source: Soc Psychiatry Psychiatr Epidemiol. 2024 Apr 5;59(12):2203–13. doi: 10.1007/s00127-024-02659-5 (PMC11522157; doi:10.1007/s00127-024-02659-5)
Supplement: Supplementary file 1 — Supplementary file1 (PDF 166 KB) [file 127_2024_2659_MOESM1_ESM.pdf]

Supplementary Materials

Impact of the COVID-19 Pandemic on Young Adults' Mental Health and Beyond:
A Qualitative Investigation Nested Within an Ongoing General Population Cohort Study

Anna Wiedemann[1-3], Peter B Jones[1-3] & Anne-Marie Burn[1-3]

[1] Department of Psychiatry, University of Cambridge, UK
[2] Cambridgeshire and Peterborough NHS Foundation Trust, UK
[3] National Institute for Health Research, Applied Research Collaboration, East of England, UK

Corresponding Author: Anna Wiedemann, Department of Psychiatry, University of Cambridge, Douglas House, 18B Trumpington Road, Cambridge, CB2 8AH, United Kingdom,
Email: aw778@medschl.cam.ac.uk

Supplementary Materials 1a

NSPN COVID-19 Interview Guide

**Introduction**	Welcome participant and introduce yourself. Thank the participant for taking part.
**Structure**	Explain set up and length. Remind participant that the interview will be recorded for transcription purposes, helps us analysing the data. Transcripts will be pseudonymised by changing their name and any identifying details of people or places mention. Notes may also be taken.
**Consent**	Reassure that participation is voluntary. There is no pressure to answer questions and there are no right or wrong answers. They can also take breaks if they wish or end the interview entirely without giving a reason.
**Confidentiality**	Interview content is confidential and will not be disclosed to anyone outside of the team unless there are concerns about their safety. Reassure them that you are in a closed room where no one can hear them. Ask them whether they are in a quiet comfortable place where they can talk with no interruptions.
**Study Purpose**	Briefly re-introduce NSPN study; explain the purpose of today's interview and that we will be asking them about their mental health-related experience during the COVID-19 pandemic. Mention that we are particularly interested to understand what things they think have made their mental health better or worse, and if there are any activities or behaviours, they found helpful to cope and maintain their mental wellbeing in that time.
**Questions**	Ask whether there are any further questions before starting.
**Recording**	Inform them that recording is going to start now.
	**START RECORDING!**
**Interview**	See topic guide on next page.
	**STOP RECORDING!**
**End**	Thank them for their participation. Inform them that they will receive their £25 gift voucher via email. Explain timeline and output of the study and how access to results will be provided. Get them back to "day-to-day" life, e.g., asked them whether they have anything nice planned for the rest of the day.
**Post-Interview**	Check and download audio recording from Zoom. Delete recording from platform. Make back-up copy of audio recording on external drive. Make general notes about the interview.

NB: The interview guide gives an indication of the type and range of questions which will be covered in the interview. It is not a script, and the discussion will develop in response to the participant's contribution and will pick up and explore issues of particular relevance and salience to each case. The wording and direction of questions will be tailored to individual participant's circumstances and phrased sensitively and appropriately according to context.

Supplementary Materials 1b

NSPN COVID-19 Topic Guide

**Contextual Background** (5 mins)	Before we start, it would be great if you could tell me a little bit about yourself.<br>*NB: Try to retrieve some contextual information about the participant, e.g., family, friends, work/study to get the participant talking and to feel comfortable.*
**Mental Health** (10 min)	How have you felt during the initial outbreak of the COVID-19 pandemic?<br>How do you feel now?<br>How has your mental health and wellbeing changed compared to before the pandemic?<br>• Is there anything you struggle with now that you didn't before?<br>• Is there anything that you now find easier that you didn't before?<br>Is there anything else about changes in your mental health and wellbeing, good or bad, since the start of the pandemic that you would like me to know?
**Relationships** (5 min)	How has the pandemic impacted your relationship with your family and friends?
**Coping & Adaptation** (10 min)	What things have you tried to maintain or improve your mental health and wellbeing since the start of the pandemic?<br>*[...] some people may have started new hobbies, or other activities to help their mental health.*<br>What would you have liked in terms of support? What do you think would have helped your mental health?
**Work** (5 min)	Can you tell me a little bit about your job/studies and how working/study arrangements have changed since the start of the pandemic?<br>How have changes in your working/study environment impacted your mental health and wellbeing?<br>How have changes in your working/study environment impacted your ability to focus or concentrate and to get things done?
**Other**	Is there anything else you would like me to know that we haven't talked about yet?

Supplementary Materials 2

NSPN COVID-19 Final Coding Framework

NB: Overarching theme titles will not be used as coding labels; they are used to group subsequent labels. Each label will be represented as column and each respondent as row in the final coding framework; respondent quotes will be summarised, and example quotes will be used instead.

Background

Any relevant background information will be summarised to enhance contextualisation and interpretation of findings. This will include basic socio-economic characteristics based on information provided in the NSPN COVID-19 Follow-up Survey between July - October 2022 as well as NSPN baseline data including NSPN ID, age, gender, ethnicity, and index of multiple deprivation. It will also include information about the current and past living situation where known as well as a brief summary about participants' work and educational background.

1. Pandemic General Impact

This theme will be used to code the wide-ranging general effects of COVID-19 based on the below-described labels; these can be both positive and negative.

1.1 Initial Impact and Response - any personal experiences related to the initial COVID-19 outbreak; this may include feelings of uncertainty or excitement, or shifting perceptions about the severity of the outbreak, but also worries about catching the virus.

1.2 Impact on Cultural and Societal Behaviours and Values - any pandemic-related impact on cultural or societal behaviours and values such as shifts in public trust and compliance, or perceived changes in mental health stigma.

1.3 Other - any other more general pandemic-related experience that is not covered by the above-mentioned four labels.

2. Social Networks & Dynamics

This theme will be used to code positive or negative experiences related to social support systems such as family, friends, or the community that occurred during the COVID-19 pandemic.

2.1 Family Life and Relationship Impact - any positive or negative aspects related to family and partner relationships or family life in more general; this could include mentioning a supportive family environment, or a strong bond with one or more family members, but also relationships breaking apart, abusive behaviour from family members, avoiding family members, or a more general lack of support.

2.2 Friendship Impact - any positive or negative experience related to friendships; this could include regular contact with friends, feeling supported by a network of friends, meeting or visiting friends, or feeling that relationships became stronger, but also losing friends or struggling to maintain friendships throughout the pandemic.

2.3 Other - any other experiences related social support systems that is not covered by the above-mentioned three labels.

3. Mental Health & Wellbeing

This theme will be used to code any aspects of mental health and wellbeing during the COVID-19 pandemic, however, some information about pre-pandemic mental health and other health issues may also be included in this theme.

3.1 Mental Health History – any aspects related to pre-pandemic mental health such as a known mental health disorder, but also undiagnosed mental health issues shall be included. This label shall be used for contextualisation of subsequent labels.

3.2 Mental Health Challenges – any aspects that pose mental health challenges during the pandemic whether or not directly related to the pandemic; this could include acute mental health problems, mental issues that interfere with day-to-day activities, suicidal ideation and behaviour, or supporting someone with mental health problems.

3.3 Access to Mental Health Support and Therapy – any aspects related to seeking help or support for either their own or someone else's mental health issues, or any aspects related to receiving treatment for mental health issues during the pandemic; this could include trying to seek support through schools, universities, or charities, contacting GP, trying to access talking therapies.

3.4 Coping Strategies and Adaptive Pursuits - any positive or negative cognitive and behavioural strategies; this could include adapting a positive attitude, taking active measure to look after oneself, keeping up with hobbies, or finding new ones, but also includes substance misuse, or social withdrawal.

3.5 Reflective Growth and Resilience - any aspects mentioned that demonstrate capacity to withstand or bounce back from adversity such as a feeling of growth throughout the pandemic.

3.6 Other - any other aspects related to mental health and wellbeing not covered by the above-mentioned labels.

4. Work & Education

This theme will be used to code both work as well as educational experiences throughout the COVID-19 pandemic. Some NSPN participants were still at university at the time of the interview, however, the majority of participants were in some form of employment at that time.

4.1 Work Challenges and Ramifications – any aspects related to both positive and negative experiences at work whether or not working from home; this could include increased stress or workload, career changes, being placed on furlough, but also things like work-related opportunities for growth.

4.2 Workplace Culture and Support – any aspects related to both positive and negative experiences about the organisational culture, values, and support systems; it is distinct from the previous label as it focuses on cultural and support aspects rather than specific challenges and consequences related to work tasks and responsibilities; this could include things like the degree of flexibility in work arrangements, support, or lack thereof, by management and colleagues.

4.3 Working From Home – any aspects related to working remotely during the pandemic; this could include both advantages and disadvantages of remote work such as impact on work-life balance, productivity, or impact on concentration and focus.

4.4 Education and Teaching – any aspects related to being a student in an educational setting during the pandemic; this could include things like the impact of remote or hybrid learning, challenges faced by students adapting to new teaching methods, or the availability of resources needed for remote learning.

4.5 Other – any other aspects that relate to work and education that are not covered through the previous labels.

5. Other

This theme will be used to code any other information of relevance that does not fit in any of the other themes; compared to the other overarching themes this theme will actually be used as a label; the framework may be amended based on themes emerging within this label.

Supplementary Materials 3

Extended Results & More Quotes

Domain 1: Pandemic General Impact

Initial Impact and Response

Grasping the Seriousness of the Situation: Initially, most participants reported being unconcerned about COVID-19. However, many reported experiencing a significant perceptual shift, often tied to a certain event such as university closures, work meetings, seeing security guards at their local supermarket, or hearing about people they knew passing away or being hospitalised.

> “ *I think when you have your own friends saying, my family member just passed away, or your friend's friend is in hospital, I think that's when you know it's like, okay, it's close to home. Like, it's not just behind the screen; it's like in your own circle. I think that's when you're just like, okay, it will affect us.* ”
>
> P20, Male, 24 years

Most participants were not too worried about catching the virus but rather about spreading it. Very few participants, reported not being worried at all. Some said they adopted a wait-and-see approach and tried to look at it from a more logical standpoint.

> “ *It didn't really worry me. I know it was killing a lot of people but I'm quite a practical person and I knew from a logical standpoint I'm young and then we did the lockdown. I was like, well, great, we're all in lockdown. We're not going near each other anyway.* ”
>
> P21, Female, 28 years

Feeling Contradicting Emotions: Despite reporting feelings of worry and anxiety during the initial outbreak, many described also feeling a sense of excitement and curiosity. One participant recalled contemplating the possibility of the government employing the army to go out on the streets, another likened the atmosphere to that of a power cut.

> “ *There was a slight element of like, you know, this is relatively new. It's kind of like when there's a power cut and everyone starts lighting candles, it's like slightly exciting for a short period of time. But I think there was a lot of anxiety, a lot of nerves (...)* ”
>
> P17, Male, 27 years

Rolling Media Coverage: Many participants mentioned feeling stressed by the rolling media coverage. A few participants reported that they stopped paying attention because it was causing them significant stress, with some describing this as a liberating experience. One participant, for instance, further reflected on the media's power in elevating levels of panic and distress, others described that the pandemic has changed their relationship with the media, making them feel more attached to it than ever before.

> “ *One thing that is probably quite significant is that I wasn't really engaged with media [before the pandemic]. The news and stuff. I would occasionally watch it. And since the pandemic, even up to now, I feel like I'm really attached to it.* ”
>
> P30, Male, 33 years

Impact on Cultural and Societal Behaviours and Values

Political Division and Distrust: Several participants expressed a growing sense of pessimism about the future. They felt frustrated with what they perceived as a lack of seriousness in the government's handling of the pandemic. Additionally, some participants noted an increase in political division within the country. Many mentioned feeling more sceptical about the government and harbouring a sense of distrust as they contemplated the future.

> “ *I would say there's just a lot more political division. So, when I meet new people, sometimes I find that they might disagree with me on certain topics and that makes it a bit awkward.* ”
>
> P26, Female, 28 years

> “ *The government wasn't taking it like the least seriously. So, it's like, it's very hard to action yourself and behave in a way when your own government aren't taking it seriously.* ”
>
> P20, Male, 24 years

Normalising Mental Health Discourse: Amidst their concerns about the future, participants also highlighted a positive change in societal attitudes. A few mentioned that they perceive speaking about mental health issues as more normalised now, with the pandemic having a positive impact on reducing mental health stigma.

> “ *I talk more about my mental health than I ever have done, not that I'm not aware of like the stigma of mental health, you know, particularly with men, but I found that when I started talking about it, like it was like people started talking about it to me more. It was like this wonderful door had been opened.* ”
>
> P16, Male, 30 years

Domain 2: Social Networks & Dynamics

Family Life and Relationship Impact

Growing Closer to Loved Ones: Almost half of the participants reported living with or moving back in with their parents for various reasons during the COVID-19 pandemic. Many were still living with their parents at the time of interview, i.e., more than two-years after the initial outbreak. Most participants shared that the pandemic has brought about a positive impact on their family and intimate relationships. They expressed feeling closer now than before the pandemic. Some participants revealed a deep sense of the preciousness of life, as they were constantly confronted with news of death, prompting them to cherish and spend more time with their loved ones. Others expressed a conscious effort to repair broken relationships as a result of this realisation. Many participants lived with their parents, and sometimes siblings, during the pandemic. The prevailing response from these individuals was a genuine sense of gratitude for the precious moments spent with their families during such challenging times. The feeling of closeness to family members was not limited to those living together; it was an equally shared sentiment among those living apart. Many participants reported increasing their contact with family members, often driven by a heartfelt desire to ensure their loved ones' well-being.

> *In terms of I guess my family we definitely got a lot closer. I actually really appreciate the time that probably I never would have gotten if I had not gone through the pandemic. That was a big positive thing, I got to spend a lot of time with my dad and my mum. I got to see my nephew basically grow up really closely.*
>
> P19, Female, 25 years

> *It's definitely made me appreciate my family more. Understanding, particularly my sister, we definitely grew a lot closer during the pandemic. And it's something that I'm actually really grateful for coming out of the pandemic.*
>
> P09, Female, 31 years

> *I got closer with my nan and grandad, seeing a lot of people dying on the news from Covid made me kind of realise that they're not gonna be around for many more years. So, I've spent a lot more time seeing and spending time with them.*
>
> P07, Male, 25 years

Likewise, individuals in existing relationships expressed that the pandemic has brought them closer to their partners. Numerous participants likened the experience of navigating the pandemic together to a relationship test, and the shared journey has become an affirmation that, in most cases, has fortified their bond and made them stronger.

> *So, it's definitely made me closer to my partner, because that was quite a big test suddenly being home 24/7 and I just found it a huge comfort. We got on really well and it was really nice, and I really enjoyed spending that much time together.*
>
> P25, Female, 28 years

Some participants initially encountered challenges and questioned whether their relationship would withstand the trials of the pandemic. Nevertheless, in numerous instances, these couples emerged even stronger.

> *And then it's just like obviously the first three, four weeks when we got together thinking, is it even going work? Because you know, when you're just in that same house, in the same area and there's just that room that, yes, one room, sometimes you're just getting frustrating like at - it was getting to a point where, are we really meant to be like together? (...) but we just made it work and then, yes, just got strong.*
>
> P13, Male, 32 years

Not everyone reported feeling closer to their families, especially those with family members living abroad. For these participants, their relationships remained largely unchanged, as their regular modes of communication and interactions persisted. In very few cases communication worsened.

Navigating Conflict: Some participants reported increased conflict with a specific family member or their partner. Whilst some successfully resolved these conflicts through increased communication, others described internalising their frustrations, often distancing themselves from these individuals.

> *I actually share a room with my older brother, he's like 27, going on 28. So, I mean like the one negative effect that I can say I had from Covid was, I started to feel like - because it's two grown men sharing a space - (...) a lot of spite started to grow because I'm home a lot, he's home a lot and I'm probably like, I don't like confrontation, especially with my brother. But I did start to feel like a lot of like bitterness like a bit of hatred inside, like a lot of spite.*
>
> P20, Male, 24 years

Several participants reported increased conflict due to differing political opinions within their families, particularly related to the COVID-19 vaccine. In many cases participants said they avoided the topic. A few went a step further and decided to keep their COVID-19 vaccination a secret.

> *My relationship with my family at the time I don't think it changed at first, because we were all on the same page about Covid. When the vaccines came out, that's when we started to have some issues because I was pro-vaccine. A lot of them weren't. So, there have been issues about that, we don't discuss it now. Yes, it bothered me a lot when some of them refused to take the vaccine.*
>
> P26, Female, 28 years

> *But one thing is that to this day, I've not actually told my dad, I've taken the vaccine. Because I know he's got a strong opinion about it. I know if I was to say it to him, then he wouldn't like that at all. So, I know to this day he thinks I haven't taken the vaccine when I actually have. My mum and sister know about it, but I never told him about it.*
>
> P28, Male, 24 years

Notably, conflicts regarding pandemic-related restrictions or the COVID-19 vaccine were not limited to family members but also arose among friends. However, participants characterised these conflicts as less impactful, with limited implications for their friendships.

Friendship Impact

Refining Friendships: Many participants found themselves growing closer to their best friends while simultaneously experiencing a sense of distance from more peripheral friendships. A small number of participants re-evaluated their friendships more deliberately, distancing themselves from those who did not value their friendship appropriately. Nonetheless, despite these changes, many described the impact of the pandemic as positive, with an overall improvement in quality of their relationships.

> *But, yes, I think the key that came out of lockdown was that I kept the friends that were willing to stick by me and didn't with the others. And I think that's very good. And it's highlighted who's actually got my back kind of thing. Who have I got as well.*
>
> P07, Male, 25 years

> *I think the main difference for me is that the pre-pandemic and post-pandemic me is that the friendships are stronger. I've had the same friendships but they're much stronger, they're much more unconditional.*
>
> P16, Male, 30 years

A few participants further reported re-connecting with old friends they had lost touch with previously. While a few participants reported losing friends and having a smaller friendship circle coming out of the pandemic. Nevertheless, they found that their closest friendships had been consolidated.

Difficulties Restoring Friendships: Some participants mentioned that not being able to share important milestones with their friends during the pandemic made it harder to maintain them and regain their pre-pandemic closeness. For some, this was due to the transition from university to work, whilst others felt more distanced from their friends because they could not be there for significant life events such as starting a family. Some participants found it challenging to have positive

conversations with their friends during the pandemic as they could not form new experiences together, leading to strain on their relationships. Even though some relationships improved, others were showing signs of fading away.

> *I think during the pandemic they - so a lot of my friends matured to a point where they were concentrating on their own families or starting families, which was really lovely, but obviously we couldn't see or be a part of that journey. So, I would say my relationship with friends is probably worse in ways.*
>
> P29, Female, 30 years

Social Life after Lockdown

Many participants found it difficult to socialise when lockdown restrictions eased, but most said these struggles were short-lived, and they soon felt more at ease. Some mentioned that observing others out and about during initiatives like the ”eat out to help out” scheme, which offered discounts on food and drinks to encourage dining out and support the hospitality industry, played a role in boosting their confidence to engage in social activities again.

> *I guess over lockdown, even without realising it, probably do lose some of that social skill. Just not seeing people as much and going back to obviously not having so much face-to-face interaction, yes, I guess some of that was lost. Which has - yes, which has grown again since coming out of that.*
>
> P12, Male, 25 years

A small number of participants described finding socialising challenging even after all restrictions have been lifted in the UK. A few participants said they are still anxious. Others reported that they either lack the desire to be around others or prefer to stay home, and as a result, some felt that the pandemic has greatly impacted their social life.

> *Even to this day, I still feel a bit anxious about going out as a result of being stuck inside for so long, especially while pregnant. So, yes, I just - I think I just tried to stick really rigidly to the rules so that we would sort of be safe.*
>
> P01, Female, 30 years

> *I started going out a bit more but then obviously as you're out, you just want to go, come back home, do nothing. But yes, that's literally, that's that what pandemic has done to me over the past two years, it's made me want to stay at home rather than go out. So that is, it's finished the social aspect of my life. Honestly, even if I'm out, I'm thinking in my mind, I'd rather be at home watching TV or on my sofa. Right? I was never that sort of person but in the past two years that's what I've become and I'm pushing people away.*
>
> P13, Male, 32 years

Domain 3: Mental Health & Wellbeing

Mental Health Challenges

Struggling with Isolation and Uncertainty: Most participants reported experiencing some kind of mental health challenge, particularly in the early stages of the pandemic. Initially, many felt anxious and isolated, with pandemic-related changes amplifying a sense of solitude. Some participants mentioned that even when they tried to keep busy, they still felt a lingering sadness. Others noted that staying at home made them think and worry more than usual.

> “ *You couldn't do much, all you would do was just at home, home, home and then that is literally it. Yes, it's just wasn't good mentally and physically and then I don't know. Yes, that was a mental aspect of it and I honestly just made you think a lot more.* ”
>
> P13, Male, 32 years

Quite a few participants described the pandemic as a source of unprecedented stress, particularly at the beginning, exacerbating anxiety levels, even among those who previously identified as calm. For some this stress manifested in weight gain and heightened health concerns. A few individuals reported struggling with uncertainty, leading to feelings of stagnation and hopelessness.

> “ *I kind of like gave up if that makes sense. So, like let's say my goal was, I don't know, go travelling. I just felt like, no this isn't something that's possible, I'm so behind. I can't get to this point. I want to learn how to drive, I'm just like, what's the point of learning how to drive. Just kind of like those negative feelings that you can't achieve something anymore.* ”
>
> P19, Female, 25 years

Some participants described a significant change in their comfort behaviour with a newfound preference for staying at home, a shift they attribute to their pandemic experiences, in particular isolation. At the time of interview a small number of individuals reported feeling overwhelmed and less resilient when faced with the prospect of re-engaging with the outside world, i.e., more than two years after the initial outbreak of the COVID-19 pandemic. For related information see Domain 2, Socialising after Lockdown.

> “ *And now if I - if it's up to me, I'll just stay at home which isn't really like me. I think that's from Covid, from being redeployed to working from home and then being home on maternity leave which I've sort of got used to just being at home with my daughter by myself. And feeling overwhelmed when I have to go out and not feeling like I could cope maybe the same way I used to if things don't quite go right.* ”
>
> P01, Female, 30 years

Furthermore, a few individuals described persisting mental health challenges such as experiencing fluctuating emotional states since the onset of the pandemic. Others expressed uncertainty about fully recovering from the pandemic's lingering effects.

> “ *I went through a few phases, a few ups and downs I would say where I've gone into a phase of apathy almost. Like, almost feeling a sense of - I just feel depressed right now and then it takes a couple of days and then I lift myself back up. But I think that's something that started with the pandemic, and I noticed hasn't completely gone away, I'd say. It hits me from time to time.* ”
>
> P08, Female, 33 years

> *Now I'm developing into a sort of stressed adult that needs to create a better work life balance (...) But I don't know, I think even with a work life balance, I don't know if it'll completely get rid of all the side effects that a pandemic has had.*
>
> P23, Male, 24 years

Aggravation of Prior Mental Health Issues: For those with pre-existing mental health conditions the pandemic intensified their struggles, adding more stress to challenges they were already facing. One participant, who consistently felt isolated before the pandemic and had difficulties forming friendships, experienced a severe emotional decline during this period.

> *I was breaking down in every possible way that I can think of. I would feel constantly down. I would feel let down and lonely and unwanted, unloved. With that I did find it that I would struggle more to take care of my daughter.*
>
> P05, Female, 27 years

Some participants noted that their social anxiety worsened due to the limited opportunities for interactions. One participant described returning to past coping strategies, such as therapy worksheets, but this proved unhelpful. Another participant reported that their mental health sharply declined during the second lockdown, a deterioration triggered by witnessing the struggles of others and feeling personally helpless, ultimately reaching a crisis point.

> *So, pre-pandemic if I was feeling that way, I could go for a walk and take myself away from everybody and maybe like emotionally regulate that way. So, I could have that cry, and nobody had to see it, but when we were all stuck at home during Covid, and you couldn't bloody go anywhere. So, I think, yeah, I think that I'm honest enough to admit that those big tear fits in front of the kids and losing my temper and everything else that triggered the lows, I think when you recognise that other people are having to see that and you can't just take yourself away, then that was a big turning point. Because it's moments like that you actually think, I need to sort out, this isn't healthy.*
>
> P29, Female, 30 years

Looking after Someone with Mental Health Issues: Those looking after someone with mental health problems reported a significant strain on their own wellbeing. One participant described balancing work with caring for their partner, finding the pandemic inadvertently masked their struggles but left them feeling increasingly delicate and on edge. Another participant highlighted the emotional and practical burden of caring for their partner, emphasising this as their most substantial indirect impact from the pandemic.

> *I was trying to be as strong as I could for the last couple of years. Trying to be something stable for her to hold on to. I've just felt like a little bit more, I don't know, like mentally a bit more on edge and a bit more delicate the last couple of months. I don't really know how to describe it other than like, I don't know feeling upset more easily, getting angry at least internally a little bit more easily.*
>
> P06, Male, 29 years

> *He was at that stage where he was one of those people that was just in bed and couldn't really do anything, wouldn't clean, wouldn't cook, just all of the hygiene goes out the window. He was not very much in survival mode. So, there was a huge demand on me for about a year. So that was probably the biggest impact for me secondary in a way.*
>
> P21, Female, 28 years

Dealing with Distanced Illness and Farewells: A few participants experienced the loss of a family member or a friend during the pandemic. Some reported seeking therapy to deal with these experiences, whilst others described the significant emotional impact not being able to visit or say goodbye due to pandemic-related restrictions.

> “ *So, that was kind of like the culmination of 15 months of not seeing someone, one of the most important women in your life and then she's got stage 3 cancer, that's really kind of - because you automatically think stage 3 cancer is not good. Any kind of cancer is not good, but stage 3 is like that's nuclear at that point. And 15 months of not seeing your own mother, it feels like I've been robbed of that time by something that's not in my control.* ”
>
> P16, Male, 30 years

COVID-19 Infection: Participants' experiences with COVID-19 infection varied. Upon testing positive for COVID-19, many individuals reported feeling initial intense fear and anxiety, especially those with pre-existing health conditions.

> “ *My experience that first sort of 24/48 hours I was really worried because I was like well I've definitely got it there's gonna be at some stage I'm gonna get really, really sick. And I didn't. It was a bad flu experience for me. And I think I'd built it up so much in my head because of my asthma and because knowing that side of it.* ”
>
> P09, Female, 31 years

> “ *The first couple of days it hit me hard. Thinking, what if I don't make it through this? Because that's all you hearing on the news, people who've got Covid, they die. So, it was just scary that thinking, what if my partner don't make it. What if I don't make it?* ”
>
> P13, Male, 32 years

Even though the majority of participants did not report experiencing prolonged symptoms after their COVID-19 infection, those who did, faced significant consequences. One participant, accustomed to long working hours in a care setting, found that Long COVID severely hindered their capacity to work, largely due to persistent exhaustion. Another participant described the sensation of 'brain fog' associated with Long COVID, likening it to the heavy, full feeling one might experience after a night of poor sleep, but in this case, the sensation was constant and relentless.

> “ *Foggy head. Like brain fog, like almost like your head's full of heavy cold or - not a headache but just kind of like, you know, if you haven't slept well and you wake up and your head feels so heavy, it's just kind of like that all the time.* ”
>
> P16, Male, 30 years

> “ *You're interacting with the world but you're also like slightly removed from it all. (. . .) I often feel like I'm doing things and walking around, I don't know, going wherever, but I also kind of feel like in my dreamy state.* ”
>
> P11, Male, 30 years

Access to Mental Health Support and Therapy

Access to Support: Participants struggling with mental health issues and considering seeking professional help encountered significant barriers to access care. The majority reported difficulties in obtaining appropriate assistance through the NHS, with common issues including unresponsive services, difficulty securing appointments, and overwhelming waiting lists. Some, expecting these obstacles, avoided seeking help altogether.

> *I didn't really try to be honest because you know it was just can you imagine the idea of like - because the waiting lists for the NHS counselling is just ridiculous anyway and I think, you know, I would prefer that in person contact.*
>
> P16, Male, 30 years

Some participants reported a lack of options for mental health support stating that the available resources were frequently unsuitable for their situation. A few individuals highlighted the gap in semi-urgent mental health support, noting that mental health issues had to significantly deteriorate before they could access help. Even though some participants found support through charities this often fell short of the help they needed.

> *But we'd spoken a little bit about my other symptoms, and they'd recommended that I need to speak to a GP about a referral for potentially ADHD. But trying to access that is extremely difficult, unless I'm going to - I know someone who's paid to have a referral for it, and I can't afford to do that at the moment.*
>
> P04, Female, 27 years

One participant had a positive experience with their long-standing GP practice, securing prompt appointments and receiving consistent, caring communication. This participant acknowledged their situation as fortunate, contrasting it with their husband's wait times at a different GP practice.

> *I know not everyone is in the same shoes. I'm really fortunate that I have a GP surgery that I can still I could call them right now and say actually I really need to speak to somebody, and I could get an appointment today, like it's still over the phone, but it's with people that I've known and trusted for so long. So, there's other on the flip side, my husband has a different GP surgery and he'd be waiting six weeks, but I do recognise how fortunate I am, but no, I was able to straight away get access and then they would text me daily, which was really, really sweet for a few weeks and get me back the medication that I've been taking for far too long, but you know it was good. It was a really supportive wrap around service.*
>
> P29, Female, 30 years

One participant who could afford private mental health support reported easily accessing these services. This individual used a prescription app to find and connect with a private therapist after struggling to secure one through the NHS.

> *So, I have an app where I used to order my prescriptions and on the resources, it showed me - it gave some resources about private therapists because I struggled to find a therapist through the NHS. So, I was able to find one privately through that app. Yes, so it was quite straightforward.*
>
> P26, Female, 28 years

Receiving Treatment: Participants who were prescribed medication generally found it to be a stabilising and valuable component of their mental health care. Those who were able to access and receive talking therapy, mostly outside of the NHS, generally reported positive experiences. For many, therapy was a crucial factor in their recovery, helping them climb out of depressive states, gain control over anxiety and panic attacks, improve self-esteem and motivation, and foster a greater sense of self-awareness. Individuals noted the convenience and privacy of phone and online sessions as beneficial during the pandemic, as these formats allowed them to express themselves freely within the comfort of their own homes.

> *I think it helped me to not know what my therapist looked like. I don't know why, it just helps that if I bumped into on the street, she wouldn't know me, and I wouldn't know her. I like that. But yes, I thought it was - well, I felt it was a positive experience. I felt like I was able to be in my home and I was speaking to somebody in my ears, but I wasn't having to - so, I could, like, cry and whatever else I needed to do.*
>
> P04, Female, 27 years

Nonetheless, one participant reported a less satisfying experience with NHS-provided talking therapy services, mentioning concerns about the professionalism and effectiveness of the treatment, which ultimately led them to discontinue the therapy.

> *After my daughter was born, I did have some CBT because her birth was quite rough. But I didn't finish the course as it was - it was quite poor CBT, sort of having an insider insight, I was quite disappointed with it.*
>
> P01, Female, 30 years

Coping Strategies and Adaptive Pursuits

Engaging with Nature: Many participants experienced a soothing connection with their natural environment. A few individuals reported an enhanced interest in nature such as becoming more interested in birds, others highlighted the positive impact of dog walking on their wellbeing.

> *I got more interested in like birds and nature. So, I probably was winding my husband up because he'd be chatting away to me and I'd just stop, be staring at a tree, be looking at like what birds I can identify by how they look, also by their sound. Which I think overall, that was yes, quite relaxing and obviously you got an achievement you know, if I can actually hear a bird and then go, I know what that is.*
>
> P10, Female, 31 years

Many participants walked outside almost every day. Being outdoors helped them relax. Some mentioned they did not see the value of walking before the pandemic, but that changed. Now, for many, walking is an active coping strategy, even if they do it less.

> *It's like every single day, there's no day that can go by without going on a walk now, both me and my wife. It start - we were going on walks before the pandemic, but it wasn't as regular, and it definitely wasn't a daily thing. But now it's sometimes even when it's dark outside in the winter. (...) Yes, it's something that I was seeing it as a bit pointless before, but now I love it, I feel so good and refreshed after a walk.*
>
> P03, Male, 30 years

Individuals with access to gardens reported dedicating more time engaging in gardening activities. Many described how being outdoors and engaging in physical work helped them cope throughout the pandemic. Some likened the experience to a form of meditation.

> *Doing the gardening and things like that, it was just accidental. Sort of just fell into that and realised actually, it's quite soothing. Sort of like fostering plants and things like that and it's - yes, I never really considered that before.*
>
> P01, Female, 30 years

Engaging with Exercise: Exercise emerged as a significant coping strategy for a considerable number of participants during the pandemic. Many emphasised its pivotal role in preserving their overall health and wellbeing. Running was a prevalent choice, with some taking up the activity for

the first time, whilst others continued their workout routine. Additionally, home workouts played a vital role although not everyone liked engaging in exercise alone.

> *I already knew pre-pandemic that the number one thing for me individually, in terms of maintaining a healthy mindset about things, is exercise. So that's the first thing that I sorted out as soon as pandemic hit was an exercise regime because I knew that would just the be all and end all of whether or not I'd be happy.*
>
> P27, Male, 26 years

> *But then with the lockdown and all the gyms being shut and how everyone was being encouraged to have exercise and spend some time outside, I think I noticed a lot more people out running who weren't experienced runners. Then I just felt a bit more like, there's no reason to be embarrassed.*
>
> P25, Female, 28 years

Some participants reported having a hard time because they could not engage in their favourite physical activities due pandemic-related restrictions and closure of sports facilities.

> *(...) it got to a point with me and my friends, and I know other guys who would do worse would like literally have bolt cutters and break open like padlocks on the pitch just to play football.*
>
> P20, Male, 24 years

Seeking Distraction: Many participants shared a common theme of trying to distract themselves, whether intentionally or not. A few reported actively seeking distraction from the news and social media. Reported activities varied a lot but quite a few individuals were eager to explore new activities or rediscovered past interests.

> *I've found crochet really helpful because it gives my hands my something to do and I can - like it gives me something to concentrate on because I'm not good enough do it without thinking.*
>
> P01, Female, 30 years

> *I didn't start drawing because I thought it would help but I was really surprised that it was helping in the end.*
>
> P18, Female, 34 years

> *I rediscovered the joy of reading, the joy of literature, fiction or literature, not the academic literature, and that was a really great joy.*
>
> P24, Female, 32 years

A few people mentioned playing computer games more often, saying it helped them take their minds off things and connect with others.

> *It's bizarre how games can actually almost, it sounds really bizarre, teleport you out of this world. And you can kind of get lost in them for a bit.*
>
> P07, Male, 25 years

Seeking Connection: Almost everyone talked about the importance of seeking social connection as a way to cope. Many discussed how they made a deliberate effort to stay in touch with others, and those living with family, partners, or children expressed how their relationships played a crucial role in maintaining their wellbeing. Some felt that being alone would have made their struggles

much harder. A few participants also highlighted the significance of their bond with pets, as it offered both companionship and the physical touch they were lacking. For more comprehensive insights into the effects of the pandemic on social networks and dynamics, see Domain 2.

Shifting Focus and Positive Thinking: Quite a few participants recognised their attempts to maintain a positive outlook. Those individuals also reported finding it helpful to centre their focus on short-term objectives and the things that were under their control. Whilst some regarded themselves as naturally positive, others made a more deliberate effort to direct their attention towards the positives in their surroundings.

> “ *So, it was just coming through the other side, you're able to just resonate on that fact that actually you've got so much to be grateful and happy for. It's okay that you're not happy all the time, but just try and concentrate more on the positives rather than the negatives of life.* ”
>
> P29, Female, 30 years

> “ *My elderly grandparents will be fine. My parents will be fine. I switched to working from home. We put all the PPE in place, for the staff. So, I was just a bit naïve, just quite positive thinker. I don't want to get bogged down in the negatives.* ”
>
> P21, Female, 28 years

Negative Coping Mechanisms and Struggles: A small number of participants reported resorting to maladaptive coping strategies, including turning to substances like alcohol and nicotine. Some faced challenges maintaining their routines, others found it difficult to manage their screen time.

> “ *My housemate has these little vape thingies. So, I tried out and I was like, oh, this is actually helping me with my stress, because it gives you like a little bit of a nicotine boost. So I've got reliant on that and then realise I know I'm using this to avoid my stress and stuff.* ”
>
> P23, Male, 24 years

> “ *I don't think I actually did a routine. I had nothing to do in the house. All I did was watch television shows and just talked to my family and played some games. Honestly it was just get up, get up whatever time you want, go sleep whatever time you want. I couldn't really establish something to do every single day.* ”
>
> P19, Female, 25 years

> “ *I'm a lot more on my phone. So, I would say I struggle with that sort of addiction to technology, I guess. Which I don't think was really there before. I also noticed how that does impact my overall health.* ”
>
> P30, Male, 33 years

Reflective Growth and Resilience

Bouncing Back Stronger: Quite a few participants described a sense of increased strength as a result of the pandemic. Even though some reported facing initial challenges, they thought the pandemic helped them grow personally, making them more resilient. Some individuals acknowledged they needed time for a shift in perspective. However, many shared that the pandemic gave them plenty of time to think about themselves and their lives, describing this process as an important

contributing factor to their personal growth. Even those who admitted to feeling slightly worn out now, acknowledged how the pandemic has helped them to become stronger.

> “ *Whilst I feel at the moment slightly like slightly worn down mentally. I don't feel I'm in a worse place. I feel in general, current events and other things notwithstanding, I feel like I have not suffered too much mentally. I feel like I've generally been growing and finding out what I want to do a bit more, which is good.* ”
>
> P06, Male, 29 years

> “ *I think during the pandemic, obviously so much was out of control, and I think for people with mental health, and I am speaking very generally, but I think you need that routine and those just daily factors that make everything similar and work for you what works and then you stick to it. So, when that control was taken away and you couldn't stick to it, I don't know. I really did drop, but I do think - so coming through the other side which is just so crazy - I'm probably the strongest I've ever been.* ”
>
> P29, Female, 30 years

Enhanced Adaptability and Awareness: Quite a few participants reported an increased feeling of adaptability, particularly in situations that do not go according to plan. Some described that the pandemic has helped them to identify other ways of coping and they feel they can face challenges more successfully now.

> “ *So, [Covid] helped me identify other ways of coping as well. So, now I feel I'm in a really good place, so if something does come up and ah, actually every day is a bit different. I feel a bit rubbish today, I kind of know, let's go do this. So, I know how to face it a bit better.* ”
>
> P18, Female, 34 years

Many further described that they now pay more attention to their mental health than before the pandemic. Some also mentioned that the pandemic has helped them learn more about mental health and understand how they react to stress. A few mentioned they have become better at recognising the signs when they get stressed out, allowing them to take appropriate action to address it.

> “ *I'm now very aware of when I struggle (. . .) rather than prior to the pandemic. If I was struggling I just thought it was a part of my day or a part of my week, my month. Whereas now if I'm struggling I can be a bit more active in improving it.* ”
>
> P09, Female, 31 years

Time-Related Adaptation Effects

A shared sentiment surfaced among several participants related to the pandemic's impact on their time perception. They noted a sense of temporal distortion, perceiving that time has passed strangely. For some, there was a sense that they had aged without realising it, holding onto the feeling that they should be much younger.

> “ *I must have turned 23 and 24 and 25 and kind of felt, felt like, how did that happen? I thought I was still 22, or 21.* ”
>
> P11, Male, 25 years

A few mentioned they believed they were not progressing as they had expected in life, feeling a sense of falling behind. Others, however, felt they had caught up since then.

> *I do feel like I'm behind in life. I think it's hard to distance the fact that we did lose these years. We didn't actually get to experience the things I think you're meant to experience when you're 21 and 22 and really getting on with your own life.*
>
> P19, Female, 25 years

> *I turned 27 at some point last year. I remember thinking before the pandemic, I was barely at 25 and I felt like it's took about a year and a half of my life and I'm never going to get that back. I was just kind of upset about that. But I feel like I've made up for that I've been meeting new people, made a lot of new friends.*
>
> P26, Female, 28 years

Domain 4: Work & Education

Work Challenges and Ramifications

Pandemic-Driven Career Disruptions: Several participants in their early-to-mid 20s highlighted missed career opportunities. One participant, for instance, expressed feeling behind in their career trajectory because of this. Quite a few participants reported that their graduate schemes were either cancelled or notably shortened due to the onset of the pandemic. For some, securing a first job post-graduation presented a significant challenge, with the tough job market negatively impacting their mental health. One participant, however, attributed their eventual employment success to the government's Kickstart Programme, which launched in September 2020 to fund employers to create jobs for young people on benefits.

> *I guess when I actually graduated I was meant to have a scheme that I was meant to go on, but unfortunately it did get taken away. So, I really had to think about what I wanted to do, which is really difficult when you're thinking about all these other things happening around you.*
>
> P19, Female, 25 years

> *I was applying for retail jobs, I was applying for jobs in my field, I was applying for literally anything that was available, and it was just a completely shoddy market. There was nothing available. People had job opportunities open just to say that they had a job opportunity open, I think. So, you'd get to the next stage, or they just declined your application from then on. It took a bit of a hit as well in terms of my mental health because it was just constant onslaught of rejections.*
>
> P23, Male, 24 years

Socio-economic Impact and Hardship: Some participants faced serious socioeconomic consequences and hardship during the COVID-19 pandemic. One participant described feeling like losing their sense of control over their life when all their work engagements were cancelled overnight.

> *I sort of lost the sense of control over my own life. And with regards to making ends meet it was just quite stressful to think like everything, all your social responsibilities are still the same, but suddenly you're being blocked from doing your work which you want to do.*
>
> P08, Female, 33 years

Whilst being furloughed (i.e., being placed on temporary leave as part of the UK Coronavirus Job Retention Scheme) was a big relief for some, most affected participants expressed concerns about job insecurity. Additionally, some struggled with the mental consequences of transitioning from an intense full-time job to suddenly doing nothing.

> *I was on furlough for eight months, I think, or something. And, yes, that was mental. It was almost a numbing sensation. You'd drive so hard, and you'd work 70-hour weeks and then you were told you've got an eight months - like a two-week break, or whatever I think it was at first (...) it was hard to understand and accept.*
>
> P07, Male, 25 years

Work Adaptations and Career Reflections: Self-employed individuals faced a tough initial period but eventually adapted to the pandemic's conditions. Notably, one self-employed participant diversified their income during the pandemic, moving away from relying solely on one source and thus feeling more resilient. Those who were furloughed or had their work hours reduced during the pandemic reported successfully transitioning back into their roles. However, for some, this process was initially challenging. Adjusting from not working, or working very little, to the demands of their jobs required effort, particularly for those who had physically demanding roles.

> *It was nice to find a new job, but I wasn't very active during the lockdown, so being on my feet for eight hours a day was really difficult. That made it harder to do the study as well, because when I'm tired I can't focus, I just want to lie down. But then eventually my body got used to working again.*
>
> P26, Female, 28 years

Because of increased time availability during lockdowns, many participants reported engaging in introspection, leading them to consider their career paths, choices, and, at times, recognise dissatisfaction with their current jobs. These realisations drove shifts in work behaviours, initiated transitions to new roles, or led to complete career changes. Some further acknowledged the pandemic's impact on mental health and how this prompted them to consider the importance of maintaining a healthy work-life balance and prioritising self-care.

> *Selfcare of mental and wellbeing, sort of really coming to the forefront and me being able to understand, okay, so actually what I was doing before probably wasn't the healthiest thing for myself. And then being able to build those habits during the lockdown. And maintain those habits as well. Now, I think I'm in a healthier place, yes, of mind and body.*
>
> P09, Female, 31 years

> *Covid gave me a lot to think about in catering. It's always been my passion, but it made think that maybe it's not 100% what I want to do if that makes sense. There's a lot of hours, a lot of stress. And having all that time off just gave me that kind of maybe I need to find something else that isn't gonna break me with all of these stressful long hours.*
>
> P07, Male, 25 years

> *I knew I wasn't happy teaching but didn't know what else to do. I think seeing the mental health impacts of Covid on children, I then ended up changing jobs afterwards. But I had a bit more time to think of it, I could see a need within a new role. I was like, actually now is a really good time for a change and I had a bit of time to think about it and think about what I wanted. I realigned some of my goals.*
>
> P18, Female, 34 years

Despite challenges, some participants recognised professional growth resulting from their work experiences during the pandemic. One participant, for instance, transitioned from a role with fewer responsibilities to becoming a team lead, navigating through the complexities of managing a team

during stressful times. Another participant, despite facing signs of burnout, acknowledged the ways in which the crisis had contributed to their career development and provided them with valuable skills and insights.

> “ *The pandemic, particularly my work, gave me an opportunity to act up as deputy service lead and grow enough to get this position and I don't know if I'd have done that otherwise.* ”
>
> P21, Female, 28 years

Workplace Culture and Support

Lack of Support and Feelings of Loneliness: Participants shared a range of experiences related to workplace culture and support, often expressing a desire for more help. Many reported feeling isolated and lonely. This feeling was especially strong for those who were starting their first jobs right after finishing university.

> “ *I was quite lonely in my first job; I was only by myself. I never got to see anybody.* ”
>
> P19, Female, 25 years

For example, one participant noted a growing sense of isolation while working from home. Their efforts to advocate for a hybrid work model as the pandemic continued, however, went unanswered. Similarly, another participant took sick leave due to inadequate support during a difficult personal period. Ultimately, both participants chose to leave their positions.

> “ *So, once I realised that even if I pushed for it, I wasn't going to be able to get into a hybrid working situation that was when I decided that I was just going to find something else that was actually with real people.* ”
>
> P26, Male, 26 years

> “ *I just had no support from work. So, in the end I just thought, you know what I'm going off sick. To be honest with you, I'd got 10 weeks full sick pay and I used, because I was just like, yes, I've given this company far, far too much. It's quite clear that if I drop dead today, no one's even come to my funeral. So, you know, it was that point that I realised I really was just a number.* ”
>
> P04, Female, 27 years

Notably, those working within the healthcare sector reported more positive experiences both in terms of support and feelings of loneliness. Many acknowledged that their ability to physically attend work acted as a protective factor for their mental wellbeing.

Lack of Effective Leadership: Several participants reported challenging relationships with their line managers or more generally management. One participant, for instance, reported that risk assessments were only addressed after a considerable delay, even though they raised concerns very early on during the pandemic. Similarly, management decisions about remote work led to conflicts and strained employee-manager relationships. For example, one participant highlighted being forced to work in the office despite the feasibility of remote work, generating frustrations within the entire team. Overall, there was a general desire for more management support during the pandemic. Participants felt management was largely inaccessible, contributing to a lack of overall guidance and assistance.

> *I would have liked more work support. I don't want to say I appreciate, because if you're in a role such as manager or whatever, you're paid to step up basically is my view. But I think the uncertainty and the stress of the pandemic made management less available to the staff in terms of one-to-one support, you know, helping people with their mental health I think. There was less availability for that on the whole.*
>
> P16, Male, 30 years

Importantly, however, not all participants shared these experiences. A few simply had full confidence in management, making them feel secure. A few others felt fully supported by their line managers, receiving flexibility and trust for independent work, but also emotional support.

> *I relied a lot actually on my manager, one of my colleagues at work. We formed a little tight knit group and so I got a lot of venting and support from them.*
>
> 21, Female, 28 years

Working from Home

Participants' experiences working from home during the pandemic varied largely, spanning strong aversion to enthusiastic approval of a fully remote setup. Both positive and negative feelings towards remote work heavily tied into their personal lives and characteristics.

At one end of the continuum, participants expressed a strong preference for working from home. They reported an enhanced work-life balance and a boost in mental wellbeing in this setting. Some participants, for instance, valued the remote work setting for its ability to reduce the culture of 'presenteeism' often experienced in traditional office environments. Rather than adhering to expectations of merely being visible in the office, they reported that working from home allowed them to concentrate more on task completion and productivity. The lack of a commute and reduced interruptions during the workday were frequently mentioned benefits, offering participants more control over their daily schedule. This allowed them to integrate activities such as meal preparation, exercise, or professional development into their day. A few individuals even attributed their career progressions, including promotions, to the effectiveness they achieved in a remote work setting.

> *I didn't have to stay behind at the office, or you know, spend extra time and it kind of gave me the freedom to prepare my meals a bit better, to actually do some exercises during the day as well. So, I got time to do more like personal development stuff as well. So, that was the happiness that stems from that.*
>
> P02, Male, 28 years

> *I found that I worked much better at home than I do in the office because I can shut myself away and really focus and concentrate. I think I always struggled with being a bit self-conscious or a little bit unsure of what I'm doing (...) Whereas, at home I feel like I can thrash things out in private and actually I find everything easier. It's given me a huge confidence bust. So, I think I've got better at my job because I've been able to have a go at things in my own time and in my own space.*
>
> P25, Female, 28 years

Some participants were more neutral about the remote setup during the pandemic. They recognised a productivity potential when working from home but were fond of the social environment of the office. One participant, in particular, expressed reservations about beginning a job in a remote

setting. They believed that their learning process was hindered due to the lack of physical presence of their team members. They felt that their progress could have been accelerated if they could have directly observed their colleagues' activities and communicated with them in person.

> "*I found that my ability to do work wasn't hindered (. . .) but my ability to professionally develop, in terms all those interactions, all that knowledge that you get from talking to people not directly about what you're working on or the work that you're doing, but just more general about what they're doing (. . .) which was frustrating because I felt that if they were there I'd be able to develop more quickly and get more engaging work.*"
>
> P27, Male, 26 years

Other participants identified difficulties in maintaining a clear boundary between personal and professional spheres, often finding it hard to disengage entirely from work. Those who adopted a hybrid working model during the pandemic valued the advantages from both in-office and remote settings. However, a few voiced apprehensions about companies possibly taking undue advantage of the remote work setup, leading to increased stress levels.

> "*I do a hybrid approach now. I go in the office two days a week. That's more for the benefit of my team, so that I'm visible. Because I recognise that I do benefit from going in a couple of days and I'm more productive in the office. I just think it builds relationships better which is better. But my gut response would be to stay at home.*"
>
> P21, Female, 28 years

On the other end of the spectrum were those who absolutely disliked working from home. For this group, the experiences included feeling a pronounced lack of support, or feelings of confinement, compounded by depressive thoughts due to monotony and isolation.

> "*It actually literally started getting depressing in the sense that it's the same four walls every day. All you're doing is sleeping, eating, working at the same location, isn't it? You're not seeing your colleagues. You're not even actually talking to your colleagues.*"
>
> P13, Male, 32 years

> "*I hated it. I hated every minute of it, I'm not going to lie. Yeah, it wasn't for me. I mean, don't get me wrong, I was lucky I still had a job, and I wasn't made for redundant. I wasn't furloughed or anything else, but my God did I hate it. It just consumed your whole existence.*"
>
> P29, Female, 30 years

Education and Teaching

Those studying during the pandemic faced diverse challenges, affecting both their academic and social experience. A prevailing sentiment was a sense of deprivation, as many missed crucial social interactions and felt they were denied a full university experience. The disruption led not only to missed internships and cancelled placements but also gave rise to feelings of frustration and anger. Many students felt isolated, struggled to stay motivated, and had difficulty maintaining focus. The lack of informal social spaces impacted relationships within student cohorts, further intensifying feelings of loneliness. Additionally, some found the monotonous environment draining their inspiration.

> *I found it really boring studying during the pandemic because I wasn't - not going to university just to sit in front of my laptop at home. Like I feel like loads of people had the same experience, so really not great just being in your bedroom, staring at a screen instead of like interacting with people.*
>
> P11, Male, 25 years

> *You don't get an outlet to release your stress, so you don't actually meet anyone or see anyone. So, it's just a constant, I've got to do work, but I can't relax because you're constantly in that environment.*
>
> P23, Male, 24 years

> *It was really difficult to focus on anything at some points, my sleeping patterns was just all over the place. There was no structure to my day. The days just melted together.*
>
> P26, Female, 28 years

Those who attended professional training courses during the pandemic voiced concerns about the potential impact of pandemic-driven changes on the quality of their education. They questioned if their modified learning experiences met the intended outcomes and worried that the quality of their training might be inferior compared to pre-pandemic standards.

> *I suppose there was this whole idea if education is so much affected by these changes, dictated by Covid, does that mean that somehow, we're not getting, that I'm not getting good enough education as I should? Am I experiencing this course the way I'm supposed to become this good clinician?*
>
> P24, Female, 32 years

> *I feel like I missed out on some stuff as well, it was a very practical course and we sort of had to muddle through and do some of that stuff online and it was a bit like - you know, in all courses, they have the learning outcomes, and I question: actually, were the learning outcomes met? I think it probably would've maybe overall diminished the quality of -yes. I'll say this diminished the quality of therapists qualifying at this time, potentially.*
>
> P30, Male, 33 years
